# Study on the Identification Methods for Effective Microorganisms in Commercially Available Organic Agriculture Materials

**DOI:** 10.3390/microorganisms8101568

**Published:** 2020-10-12

**Authors:** Ashutosh Bahuguna, Ah-ryeong Joe, Vishal Kumar, Jong Suk Lee, Sung-Youn Kim, Ji-Young Moon, Soon-Kil Cho, Hyunjeong Cho, Myunghee Kim

**Affiliations:** 1 Department of Food Science and Technology, Yeungnam University, Gyeongsan, Gyeongsangbuk-do 38541, Korea; ashubahuguna@ynu.ac.kr (A.B.); whdkfud12@naver.com (A.-r.J.); vkaggarwal180@gmail.com (V.K.); 2 Division of Food and Nutrition and Cook, Taegu Science University, Daegu 41453, Korea; jslee1213@ynu.ac.kr; 3 Experiment Research Institute, National Agricultural Products Quality Management Service, Gimcheon 39660, Korea; youn5326@korea.kr (S.-Y.K.); skc2224@korea.kr (S.-K.C.); hjcho201@korea.kr (H.C.); 4 Gyeonggi Provincial Office, National Agricultural Products Quality Management Service, Anyang 14035, Korea; jymoon76@korea.kr

**Keywords:** *Bacillus*, effective microorganisms, RAPD-PCR, species-specific PCR

## Abstract

The identification of microorganisms in closely related groups is challenging. The present work focused on the different molecular methodology for the accurate microbial identification in the five commercially available organic agriculture materials enriched with effective microorganisms. From the tested five organic agricultural materials, a total of seven distinct bacterial colonies (A-1, B-1, C-1, D-1, E-1, E-2, and E-3) were isolated and processed for sequential identification utilizing HiCrome™ *Bacillus* agar, biochemical tests with API CHB50, 16S rRNA gene analysis, random amplified polymorphic DNA (RAPD), and species-specific PCR analysis. All the isolated microorganisms were Gram-positive rods and spore former belonging to *Bacillus* group and appeared as a differential characteristic feature on HiCrome™ *Bacillus* agar. All isolates showed high-percentage similarities with the different members of *Bacillus* species in biochemical testing and 16S rRNA gene analysis. The collective identification results revealed isolates, A-1, B-1, and C-1, close to *B. velezensis*. Further RAPD-PCR and species-specific PCR discriminated and provided confirmatory evidence for D-1 as *B. thuringiensis* and E-1, E-2, and E-3 as *B. licheniformis*, respectively. In addition, presence of *B. thuringiensis* was also confirmed by toxin crystal protein staining. In conclusion, the species-specific primers could be used as a rapid and accurate identification tool to discriminate closely related *Bacillus* species such as *B. subtilis*, *B. licheniformis*, and *B. thuringiensis*.

## 1. Introduction 

In the past few decades, the frequent use of chemical fertilizers for agricultural purposes has increased drastically to meet the food demands of a rapidly increasing population. The excessive use of chemical fertilizers has an adverse impact on the agricultural ecosystem. Therefore, research has focused on alternatives to chemical fertilizers to improve crop productivity without affecting the soil health and agricultural ecosystem. Several microorganisms have proven their potential as suitable candidates for sustainable agricultural development. Such microorganisms have been intensely introduced into soil to improve fertility and plant growth [1,2] and are broadly known as beneficial soil microorganisms [3]. Recently, many studies have proposed microorganisms derived from organic fertilizers as safe and effective alternatives to chemical fertilizers [4]. 

As a pioneer, Higa (1996) [5] utilized the consortium of several natural microorganisms in a specific manner and proposed effective microorganisms for sustainable agricultural development. The effective microorganisms for agricultural purposes mainly belong to the genera *Arthrobacter* [6], *Bacillus* [7], and *Pseudomonas* [8]. Among them, several members of *Bacillus* species are attracting more attention owing to their spore-forming nature, which helps them to survive in fields for longer durations and favors their storage for commercial purposes [9]. Several *Bacillus* species are now commercially available, including RhizoVital^®^ (*Bacillus amyloliquefaciens* FZB42; ABiTEP GmbH, Berlin, Germany), Amylo-X^®^ WG (*B. amyloliquefaciens* subsp. *plantarum* D747; Certis Europe BV, Utrecht, Netherlands), RhizoPlus^®^ (*B. subtilis* FZB24; ABiTEP GmbH, Berlin, Germany), Sonata^®^ (*B. pumilus* QST2808; AgraQuest, Inc., Davis, California, USA), and Taegro^®^ (*B. subtilis* var. *amyloliquefaciens* FZB24; Novozymes Biologicals Inc., Salem, Virginia, USA) [10,11]. These species and subspecies are closely related to each other; thus, proper differentiation of these species and subspecies is somewhat difficult. 

Traditionally, biochemical tests, fatty acid profiling, and DNA–DNA hybridization have been used for microbial identification, which are very time-consuming and hence not applicable for rapid identification [12]. The next step is 16S rRNA gene sequencing, which is widely used for the molecular identification of bacteria up to the species level. However, it has the limitation of the inability to properly identify and distinguish between closely related groups, such as *Bacillus* species, where *B. subtilis* displays great sequence similarity with *B. atrophaeus*, *B. amyloliquefaciens*, and *B. licheniformis* at the 16S rRNA gene level [12,13]. For the proper and accurate identification of closely related species, the simultaneous involvement of several conventional and molecular techniques is required. 

In Korea, numerous types of commercial organic agriculture materials enriched with effective microorganisms (AMEM) are available, which typically contain *Bacillus* species individually or in combination with other effective microbial species. Although most of the AMEM-producing companies revealed the identity of the used effective microorganism, few companies did not disclose the bacterial identity. Moreover, the identity of the effective microorganism in these commercial agricultural materials is based on the old traditional classification, which needs to be reexamined owing to the recent taxonomical changes in many bacterial species, including *Bacillus* species [11]. In this respect, the present study was performed to isolate and identify the bacterial species present in five frequently used commercially available AMEM in Korea, utilizing phenotypic, biochemical, and molecular methodologies.

## 2. Materials and Methods 

### 2.1. Microorganisms and AMEM Products

The reference microbial strains used in the present study were collected from the American Type Culture Collection (ATCC), Korean Collection for Type Cultures (KCTC), and National Agricultural Products Quality Management Service (NAQS) (Table S1). Five distinct types of AMEM were purchased from different Korean companies and coded as A, B, C, D, and E. The basic constituents, supplemented microorganisms, pH, and physical state of different AMEM are listed in Table 1.

### 2.2. Isolation of Bacteria from AMEM 

**Figure 1:**
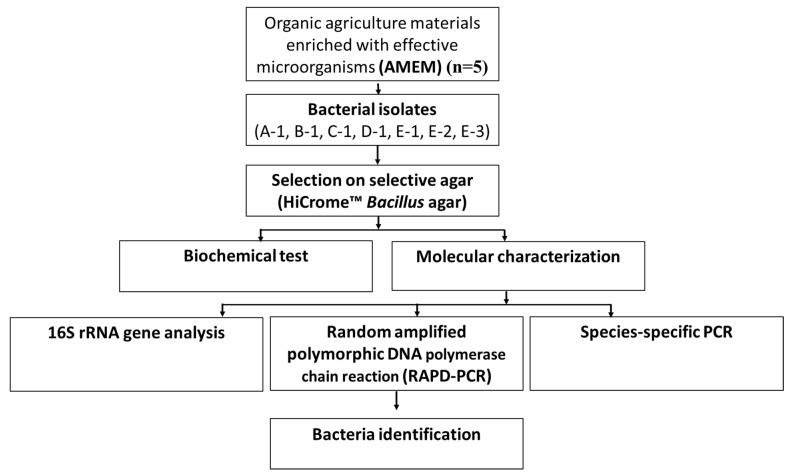
Systemic representation of bacterial identification in commercially available organic agriculture materials enriched with effective microorganisms.

Microorganisms from different commercial AMEM were isolated by serially diluting up to 10^−7^ dilutions in 0.2% peptone water. Finally, 100 µL of each dilution was spread over the agar plates with different growth media, i.e., brain heart infusion agar (BD Biosciences, Franklin Lakes, NJ, USA), nutrient agar (BD Biosciences, Franklin Lakes, NJ, USA), and tryptic soy agar (BD Biosciences, Franklin Lakes, NJ, USA). Plates were incubated at 37 °C for 24 h to observe microbial growth. All bacterial isolates were repeatedly streaked on agar plates to obtain single-cell bacterial colonies and processed for identification utilizing different methodologies as depicted in Figure 1.

### 2.3. Morphological Characteristics of AMEM Isolates

All the bacterial isolates from AMEM and reference bacterial strains were stored in 25% glycerol stock at −20 °C for long-term use. Bacterial isolates were studied for their morphological characteristics, such as colony color, colony shape, colony elevation, and colony texture [14], as well as microscopic characteristics, such as Gram reaction, shape of bacterial cells, and spore staining. 

### 2.4. Differential Properties and Biochemical Identification 

The differential properties of all the bacterial isolates from different AMEM were examined in HiCrome™ *Bacillus* agar (HiMedia Laboratories, Mumbai, MS, India). For the biochemical analysis, the single purified colonies of the isolates were suspended in normal saline solution (0.85% NaCl) to achieve a turbidity of 2 McFarland. Later, bacterial suspension was added to the different wells of the API 50CHB, and a further process was performed according to the manufacturer’s instructions (bioMerieux, Marcy I’Etoile, France). Finally, the results were analyzed using the online software apiwebTM (https://apiweb.biomerieux.com) by submitting negative and positive responses according to the reference color reading table.

### 2.5. 16S rRNA Gene Sequencing Analysis 

16S rRNA gene sequences of all the bacterial isolates were obtained by availing the commercial facility provided by SolGent Co., Ltd., Daejeon, Republic of Korea. The 16S rRNA gene region of DNA was amplified using 27F (5′-AGAGTTTGATCCTGGCTCAG-3′) and 1492R (5′-GTTTACCTTGTTACGACTT-3′) primers (SolGent Co., Ltd., Daejeon, Republic of Korea) using the PCR conditions: initial denaturation at 95 °C for 15 min, 30 cycles of denaturation at 95 °C for 20 s and annealing at 50 °C for 40 s, extension at 72 °C for 1 min 30 s, and final extension of 5 min at 72 °C. Finally, the amplified product was sequenced using BigDye^®^ Terminator v3.1 cycle sequencing kit (Thermo Fisher Scientific, Waltham, MA, USA) and analyzed on ABI PRISM 3730 XL DNA analyzer (Applied Biosystems, Foster City, CA, USA). The homology of sequences was confirmed by comparing and analyzing the base sequence of 16S rRNA gene using BLASTn.

### 2.6. RAPD-PCR 

RAPD-PCR was performed for all the bacterial isolates to confirm their identity up to the species level. Four types of 10-mer random primers were used for RAPD analysis. The primers were named as primer A (5′-GTGATCGCAG-3′), primer B (5′-CTTTCGCTCC-3′), primer C (5′-CGCAGACCTC-3′), and primer D (5′-GAACTGGAGT-3′). PCR was conducted in a reaction mixture of 25 μL, containing DNA template (1.0 μL, 50 ng), 12.5 μL GoTaq^®^ G2 Green Master Mix (pH 8.3, 1.5 mM MgCl_2_, 200 μM of each dNTP, DNA polymerase) (Promega; Madison, WI, USA), primer (2.5 μL) (Bionics, Seoul, Republic of Korea), and nuclease-free distilled water (9.0 μL). PCR was carried out at initial denaturation for 5 min at 94 °C, followed by 40 cycles [95 °C for 15 s (denaturation), 36 °C for 15 s (primer A and primer B), 38.4 °C for 15 s (primer C), or 28.1 °C for 15 s (primer D) (annealing), and 72 °C for 2 min (extension)], and a final extension at 72 °C for 4 min. The amplified RAPD-PCR product (10.0 μL) was resolved on a 1.2% agarose gel at 50 V, followed by staining with a GelRed^®^ fluorescent dye (Biotium, Fremont, CA, USA) for 10 min, and visualized using a gel documentation system (Vilber Lourmat, Marne-la-vallee, France).

### 2.7. Species-Specific PCR 

In order to identify *B. subtilis*, *B. thuringiensis*, and *B. licheniformis* at the species level, species-specific PCR was performed. The species-specific primers were designed for the *ytcP* gene of *B. subtilis* (F 5′-CTTACGGGTTATCCCGC-3′ and R 5′-CCGACCCCATTTCAGACATATC-3′) [15], *XRE* gene of *B. thuringiensis* (F 5′-AAGATATTGCAAGCGGTAAGAT-3′ and R 5′-GTTTTGTTTCAGCATTCCAGTAC-3′) [16], and *Blich* gene of *B. licheniformis* (F 5′-AKACGGAAGTGACGGGAAC-3′ and R 5′-AGAAACTTTTCRAGCGCTT-3′) [12]. The total volume of the reaction mixture for PCR was 25 μL, which contained DNA template (1.0 μL, 50 ng), GoTaq^®^ G2 Green Master Mix (12.5 μL, pH 8.3, 1.5 mM MgCl_2_, 200 μM of each dNTP, DNA polymerase), species-specific forward primer (1.0 μL), reverse primer (1.0 μL), and nuclease-free distilled water (9.5 μL). PCR was performed according to the conditions mentioned in Table S2. The species-specific PCR product was resolved on 1.2% agarose gel and visualized using a gel documentation system (Vilber Lourmat, Marne-la-vallee, France).

### 2.8. Crystalline Protein Staining

For the identification of *B. thuringiensis,* crystal protein staining was performed as the method suggested by USFDA [17]. Briefly, bacterial isolate was grown on nutrient agar medium and incubated at 30 °C for 24 h, followed by 5 days of incubation at room temperature. After incubation, bacterial smear was prepared, air-dried, and fixed by gentle heating. The bacterial smear was flooded with methanol for 30 s, followed by the addition of 0.5% basic fuchsin. Sides were heated gently until steam appeared. Finally, slides were rinsed with water and visualized under the microscope (SOMETECH, Seoul, Republic of Korea) for the presence of tetragonal (diamond-shaped) toxin crystals. 

## 3. Results and Discussion

### 3.1. Isolation of Bacteria from AMEM

From five distinctive types of AMEM, different bacterial colonies were isolated using a standard isolation method. Microscopy examination and investigation of colony morphology in solid medium and appearance in liquid medium revealed the presence of single types of bacteria in products AMEM-A, AMEM-B, AMEM-C, and AMEM-D, and that of three distinct types of bacteria in AMEM-E, which were named as A-1, B-1, C-1, and D-1, and E-1, E-2, and E-3, respectively. Gram staining followed by microscopy examination suggested that all the isolates were Gram-positive, rod-shaped bacteria (Table S3). 

### 3.2. Differential Properties of the Bacterial Isolates on HiCrome™ Bacillus agar

**Figure 2:**
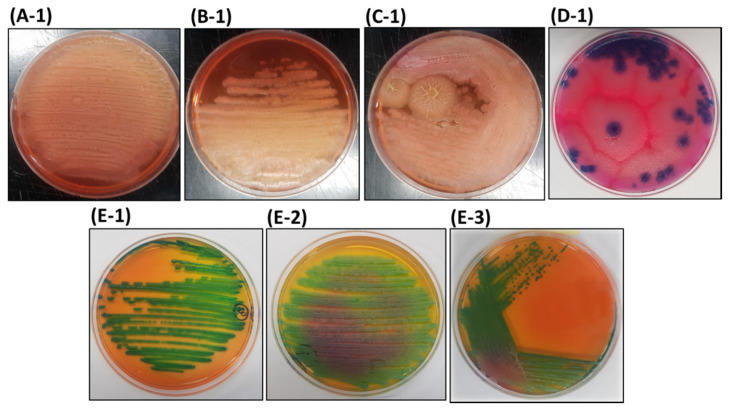
Morphology of bacterial colonies isolates from different commercially available organic agriculture materials enriched with effective microorganisms on HiCrome™ *Bacillus* agar. A-1, B-1, C-1, D-1, E-1, E-2, and E-3 are the bacterial isolates from AMEM-A, AMEM-B, AMEM-C, AMEM-D, and AMEM-E, respectively.

Most *Bacillus* spp. are known as the principal biological components used in various organic agriculture materials, owing to their stress management, disease prevention, and plant growth promotion properties [11,18,19]. The AMEM-producing company highlighted the presence of *Bacillus* species in their products, so as the first step of identification, all the isolates were grown on chromogenic HiCrome™ *Bacillus* agar, a differential media for *Bacillus* species [20]. The bacterial isolates A-1, B-1, and C-1 were white, irregular, and wrinkle shaped with yellow/pinkish-colored media pigmentation (Figure 2), which are the characteristic features of *B. subtilis* on HiCrome™ *Bacillus* agar medium [20]. However, *B. amyloliquefaciens* also displays similar culture characteristics [19]. These results provide the elementary evidence that isolates A-1, B-1, and C-1 may be *B. subtilis*, as supported by Alippi and Abrahamovich (2019) [20], who demonstrated the growth of 31 different strains of *B. subtilis* on HiCrome™ *Bacillus* agar and observed that 70.9% (22/31) of strains emerged as white, irregular, and wrinkle-shaped colonies with yellow-colored media pigmentation. In contrast, the bacterial isolate D-1 appeared as flat, large, and blue-colored colonies (Figure 2), which are the typical features of *B. thuringiensis* on HiCrome™ *Bacillus* agar [20], indicating that the isolate D-1 is *B. thuringiensis*. However, few closely related bacterial species such as *B. cereus* and *B. mycoides* also appear with similar characteristics, thus making the proper identification ambiguous.

The bacterial isolates E-1, E-2, and E-3 appeared as greenish and glistering with irregular colony characteristics with greenish-yellow media pigmentation (Figure 2), a typical characteristic of *B. licheniformis* on HiCrome™ *Bacillus* agar [20]. The culture-characteristic-based appearance of E-1, E-2, and E-3 on HiCrome™ *Bacillus* agar provided preliminary evidence that these isolates may be *B. licheniformis*. However, *Brevibacillus brevis* and *Brevi. laterosporus* also exhibit similar culture characteristics on HiCrome™ *Bacillus* agar [20], thereby necessitating the use of an additional robust methodology for accurate identification.

Colony characteristics on the HiCrome™ *Bacillus* agar provided an elementary idea about the identity of the bacterial isolates from AMEM; therefore, further detailed analysis is mandatory to provide correct confirmatory identification of the bacterial isolates.

### 3.3. Biochemical Characterization 

The analytical profile index (API) is a method for the rapid identification of microbes up to the species level based on several biochemical tests and database searches (https://apiweb.biomerieux.com). The bacterial isolates A-1, B-1, C-1, D-1, E-1, E-2, and E-3 were tested on API CH50 CHB, which is a biochemical method conventionally used for the rapid identification of *Bacillus* and related genera based on biochemical examination and fermentation of 49 carbohydrates. API CH50 CHB examination identified isolates A-1, B-1, and C-1 as *B. subtilis* or *B. amyloliquefaciens* with a high percentage similarity of 99.4%, 99.7%, and 99.4% and substantial T-index of 0.85, 0.81, and 0.85, respectively (Table S4). The findings of API were similar to those of HiCrome™ *Bacillus* agar and failed to identify isolates A-1, B-1, and C-1 as a single species (*B. subtilis* or *B. amyloliquefaciens*). Furthermore, the API detection kit identified the isolate D-1 as a *B. cereus* with 96.0% similarity and 0.95 T index (Table S4). Although the API results suggest D-1 as a *B. cereus*, the literature suggests that *B. cereus* has colony morphology and biochemical characteristics similar to those of *B. thuringiensis* [20]. Hence, further examination is required to avoid ambiguities. Further, isolates E-1, E-2, and E-3 exhibited 99.9%, 99.7%, and 99.7%, respectively, similarity to *B. liceniformis* (Table S4) and showed consistency with the findings of HiCrome™ *Bacillus* agar, although molecular characterization is required for confirmatory identification. 

### 3.4. Molecular Characterization Based on 16S rRNA Gene Analysis

16S rRNA gene sequencing is the most common method for bacterial identification [21] and is regarded as the most acceptable method for identifying genera and species for the isolates that do not fit properly in the biochemical profile of the existing system [21]. The strains with 97% sequence identity between the 16S rRNA genes are recognized as being the same species [12]. The isolated bacteria from AMEM were processed for 16S rRNA gene sequencing, followed by a gene bank database search (Table S5). More than 99% sequence similarity of A-1 (NCBI GenBank accession no. MW020274), B-1(NCBI GenBank accession no. MW020275), and C-1 (NCBI GenBank accession no. MW020276) isolates was observed between *B. velezensis* and *B. subtilis* (Table S5). Similarly, isolate D-1 (NCBI GenBank accession no. MW020277) displayed more than 99% sequence similarity with *B. proteolytic* and *B. thuringiensis* (Table S5). The isolate E-1 (NCBI GenBank accession no. MW020278) displayed more than 97% sequence similarity with *B. hyanisis* and *B. licheniformis* (Table S5), while isolates E-2 (NCBI GenBank accession no. MW020279) and E-3 (NCBI GenBank accession no. MW020280) exhibited more than 98% sequence similarity with *B. hyanisis* and *B. licheniformis* (Table S5). The results of 16S rRNA gene analysis were ambiguous and failed to distinguish the isolates as single species. The basic limitation of 16S rRNA gene analysis is its inability to discriminate closely related groups [22]. Unfortunately, a high percentage sequence homology occurred (98.1–99.8%) between the members of *Bacillus* species, thus resulting in the inability of 16S rRNA gene analysis to properly distinguish between species [12,15]. In other words, it is difficult to differentiate *B. subtilis*, *B. amyloliquefaciens*, and *B. licheniformis* using 16S rRNA gene sequencing, owing to the high sequence similarities. Therefore, another approach is required for the appropriate identification of isolates that are closely related to each other.

### 3.5. RAPD-PCR and Species-Specific PCR Analysis

**Figure 3:**
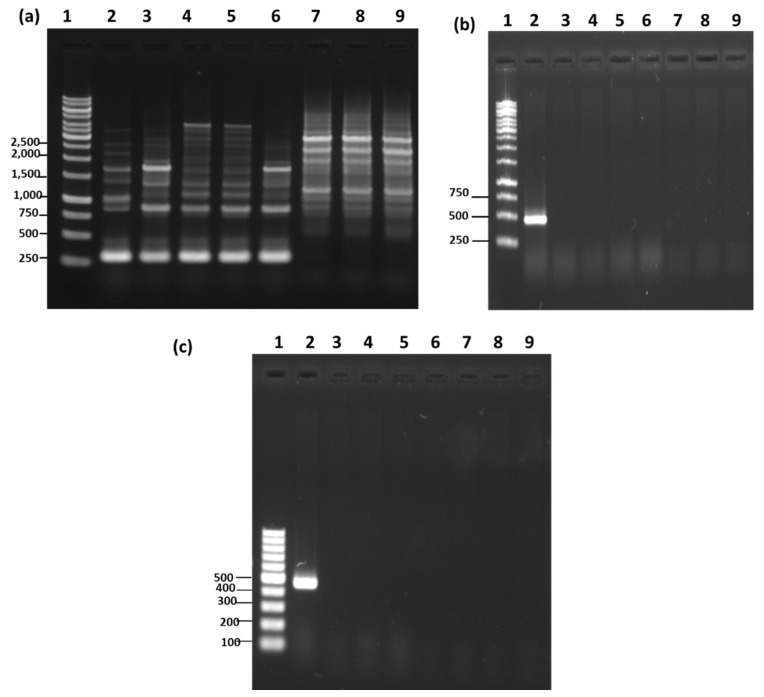
(**a**) RAPD-PCR for various reference strains of *Bacillus subtilis*. Lane 1: 1 kb size ladder (unit: bp); Lane 2: *Bacillus subtilis* ATCC6633; Lane 3: *Bacillus subtilis* KCTC3014; Lane 4: *Bacillus subtilis* KCTC3135; Lane 5: *Bacillus subtilis* KCTC2217; Lane 6: *Bacillus subtilis* KCTC3239. (**b**) *Bacillus subtilis* species-specific PCR (*ytcP* gene). Lane 1: 1 kb size ladder (unit: bp); Lane 2: *Bacillus subtilis* ATCC6633; Lane 3: *Bacillus thuringiensis* KCTC3452; Lane 4: *Bacillus megaterium* KCTC1098; Lane 5: *Bacillus velezensis* KCTC13012; Lane 6: *Bacillus licheniformis* ATCC21415; Lane 7: *Bacillus amyloliquefaciens* KCTC1660; Lane 8: *Bacillus pumilus* ATCC7061; Lane 9: *Bacillus mojavensis* ATCC51516. (**c**) Species-specific PCR using *ytcP* gene primer on microorganisms isolated from commercially available organic agriculture materials enriched with effective microorganism. Lane 1: 0.1 kb size ladder (unit: bp); Lane 2: *Bacillus subtilis* ATCC6633; Lane 3: A-1; Lane 4: B-1; Lane 5: C-1; Lane 6: D-1; Lane 7: E-1; Lane 8: E-2; Lane 9: E-3.

**Figure 4:**
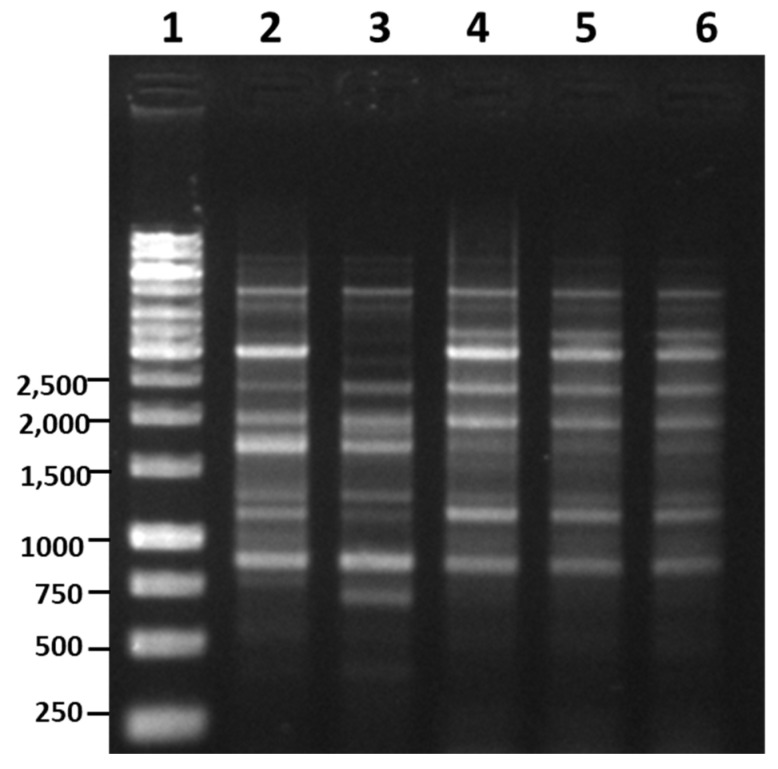
RAPD-PCR of various reference strains of *Bacillus velezensis*. Lane 1: 1 kb size ladder (unit: bp); Lane 2: *Bacillus velezensis* KCTC13012; Lane 3: *Bacillus velezensis* KCTC13417; Lane 4: A-1; Lane 5: B-1; Lane 6: C-1.

RAPD-PCR analysis has been found to be effective in generating strain and species-specific DNA amplification profiles that help in species identification [15]. In some cases, RAPD-PCR amplifies a unique species and strain-specific fragment that is further used to develop a specific primer for rapid and accurate identification [15]. In the present study, four random primers were examined for their RAPD pattern against the eight different bacteria of *Bacillus* species (Figure S1). Based on the reproducibility and the amplification profile, primer B was selected for further RAPD analysis. The first isolates A-1, B-1, and C-1, which are supposed to be *B. subtilis* (as claimed by those companies and also suggested by the earlier results), were examined for RAPD-PCR using six reference strains of *B. subtilis* (Figure 3a). RAPD-PCR demonstrated that the two reference strains, *B. subtilis* KCTC 3104 (lane 3) and *B. subtilis* KCTC 3239 (lane 6), showed identical RAPD profiling, which is very close to the RAPD pattern of the reference strain *B. subtilis* KCTC 6633 (lane 2) (Figure 3a). The remaining two reference strains, *B. subtilis* KCTC 3135 (lane 4) and *B. subtilis* KCTC 2217 (lane 5), showed identical RAPD patterns, which were slightly different from the other three reference strains (lane 2, 3, and 6) (Figure 3a). In all the six reference strains, a strong amplification of ~275 bp and ~850 bp was observed and could be utilized as a dominant marker for RAPD identification of *B. subtilis* (Figure 3a). The RAPD patterns of isolates A-1, B-1, and C-1 did not match the RAPD profile of any reference strain, thus disproving the claim that these bacteria were *B. subtilis*. To further validate the RAPD-PCR results, *B. subtilis*-specific primers were used. First, the specificity of the species-specific primer was examined utilizing eight closely related *Bacillus* species (Figure 3b). The amplification of ~500 bp DNA fragment only in *B. subtilis* (lane 2) while no amplification was observed in any of the closely related *Bacillus* species was indicative of the high specificity of the selected primer for *B. subtilis* (Figure 3b). These results are in accordance with the findings of Kwon et al. (2009) [15], who tested the same primer (*ytcP*) in 14 different *B. subtilis* strains and observed the amplification of a ~500 bp DNA fragment. In addition, when the same specific primer was tested on four different strains of *B. amyloliquefaciens*, five different strains of *B. licheniformis*, and one strain of *B. thuringiensis*, it displayed no amplification, confirming the high specificity of the primer towards *B. subtilis* [15]. Knowing the high specificity of the primer (*ytcP*) towards *B. subtilis*, we tested the *ytcP* primer against the isolated microorganism from AMEM and observed no amplification of specific 500 bp DNA fragment (Figure 3c), confirming that these isolates are not *B. subtilis*, which contradicts the claims of AMEM-A, AMEM-B and AMEM-C producing companies. One of the major reasons for this mismatch is the reclassification of several *Bacillus* species as *B. velezensis* by recent taxonomy analysis [11]. Furthermore, 16S rRNA gene analysis also suggested that isolates A-1, B-1, and C-1 were the closest homologs of *B. velezensis*; therefore, we further performed RAPD-PCR for isolates A-1, B-1, and C-1 using *B. velezensis* as a reference strain. The results revealed that the reference strains, *B. velezensis* KCTC13012 (lane 2) and *B. velezensis* KCTC13417 (lane 3), and A-1, B-1, and C-1 have similar RAPD patterns (Figure 4). A dominant amplicon of ~750 bp, ~2000 bp, and ~2500 bp was observed in all the tested reference strains and isolates A-1, B-1, and C-1, indicating that these strains were *B. velezensis*. However, further confirmatory studies are required to establish these strains as *B. velezensis*. Many recent studies suggested a high similarity between *B. subtilis*, *B. velezensis,* and *B. amyloliquefaciens* [11]. Even some members of *B. amyloliquefaciens* has been reclassified as *B. velezensis* [11]. Additionally, our API CH50B identification results suggested isolates A-1, B-1, and C-1 either *B. subtilis* or *B. amyloliquefaciens*, therefore, to rule out any ambiguity, we performed RAPD-PCR for isolates A-1, B-1, and C-1 using *B. amyloliquefaciens* as a reference strains. The results revealed that the reference strains *B. amyloliquefaciens* KCTC1660 (lane 2), *B. amyloliquefaciens* KCTC1666 (lane 3), *B. amyloliquefaciens* KCTC3002 (lane 4), and A-1, B-1, and C-1 do not have much similarities in RAPD patterns (Figure S2), suggesting A-1, B-1, and C-1 do not belong to the strains of *B. amyloliquefaciens*. 

**Figure 5:**
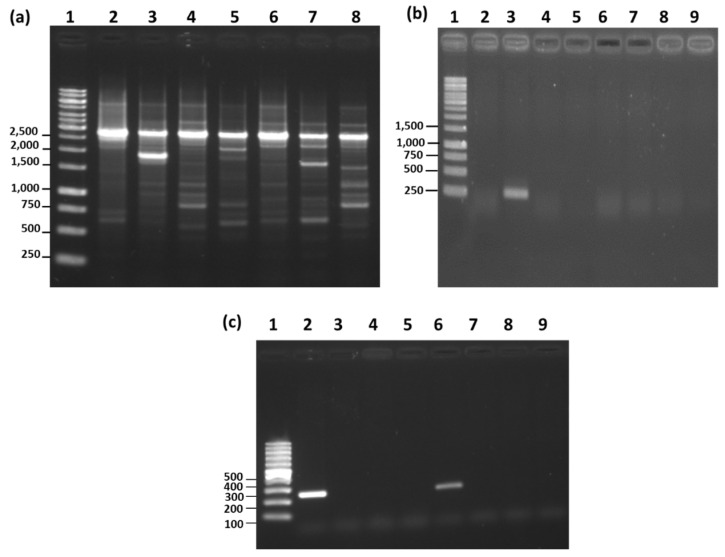
(**a**) RAPD-PCR of various reference strains of *Bacillus thuringiensis*. Lane 1: 1 kb size ladder (unit: bp); Lane 2: *Bacillus thuringiensis* KCTC3452; Lane 3: *Bacillus thuringiensis* KCTC1508; Lane 4: *Bacillus thuringiensis* KCTC1510; Lane 5: *Bacillus thuringiensis* KCTC1511; Lane 6: *Bacillus thuringiensis* KCTC1524; Lane 7: *Bacillus thuringiensis* KCTC1525. (**b**) Species-specific PCR result of pure strains *Bacillus thuringiensis* (*XRE* gene primer). Lane 1: 1 kb size ladder (unit: bp); Lane 2: *Bacillus subtilis* ATCC6633; Lane 3: *Bacillus thuringiensis* KCTC3452; Lane 4: *Bacillus megaterium* KCTC1098; Lane 5: *Bacillus velezensis* KCTC13012; Lane 6: *Bacillus licheniformis* ATCC21415; Lane 7: *Bacillus amyloliquefaciens* KCTC1660; Lane 8: *Bacillus pumilus* ATCC7061; Lane 9: *Bacillus mojavensis* ATCC51516. (**c**) Species-specific PCR using *XRE* gene primer on microorganisms isolated from commercially available organic agriculture materials enriched with effective microorganism. Lane 1: 0.1 kb size ladder (unit: bp); Lane 2: *Bacillus thuringiensis* KCTC3452; Lane 3: A-1; Lane 4: B-1; Lane 5: C-1; Lane 6: D-1; Lane 7: E-1; Lane 8: E-2; Lane 9: E-3.

**Figure 6:**
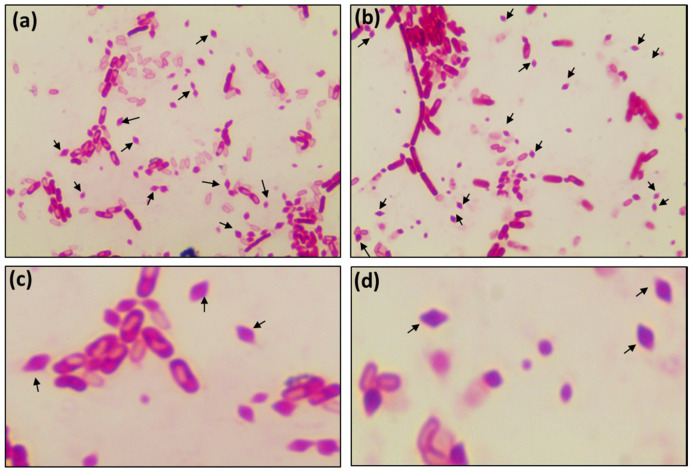
Crystal protein staining for the detection of *Bacillus thuringiensis*. (**a**) *Bacillus thuringiensis* KCTC3452; (**b**) Microbial isolate D-1 (100× magnification). (**c**,**d**) A magnified view of (**a**,**b**). Arrows show the presence of tetragonal (diamond-shaped) toxin crystals.

Next, the isolated D-1 was examined, which was claimed as *B. thuringiensis* by the AMEM-D company. Our HiCrome™ *Bacillus* agar results also suggested the isolate D as *B. thuringiensis*, and similarly, the 16S rRNA gene findings also displayed a high percentage similarity with *B. thuringiensis*. However, the API results suggested that it was *B. cereus*. To address this discrepancy, RAPD-PCR of isolate D-1 was performed using six reference strains of *B. thuringiensis* (Figure 5a). The RAPD-PCR profile suggested that all the reference strains and isolate D-1 had a dominant amplicon of ~2500 bp, indicating isolate D-1 as *B. thuringiensis* (Figure 5a). As a final confirmatory test, *B. thuringiensis*-specific PCR was performed. First, the specificity of primer (*XRE* gene) towards *B. thuringiensis* was determined by performing PCR for different *Bacillus* species strains. The results revealed the specific amplification of the ~246 bp fragment in only *B. thuringiensis*, suggesting high specificity of the primer towards *B. thuringiensis* (Figure 5b). Furthermore, the primer was tested on the isolates from AMEM, and the results demonstrated the amplification of a ~246 bp fragment in isolate D-1 and confirmed it as *B. thuringiensis* (Figure 5c). The results are consistent with the findings of Wei et al. (2019) [16], showing the high specificity of *XRE* primers for identifying *B. thuringiensis*. Moreover, it has been well-documented that *B. thuringiensis* selectively produces insecticidal crystal proteins [16,23], which can be utilized as an appropriate marker for proper identification. Herein, we examined the presence of crystal proteins using crystal protein staining and observed the presence of these crystal proteins in isolate D-1 (Figure 6). Collectively, all the results (HiCrome™ *Bacillus* agar, RAPD-PCR, species-specific PCR, and crystal protein staining) affirmed isolate D-1 as *B. thuringiensis*. 

**Figure 7:**
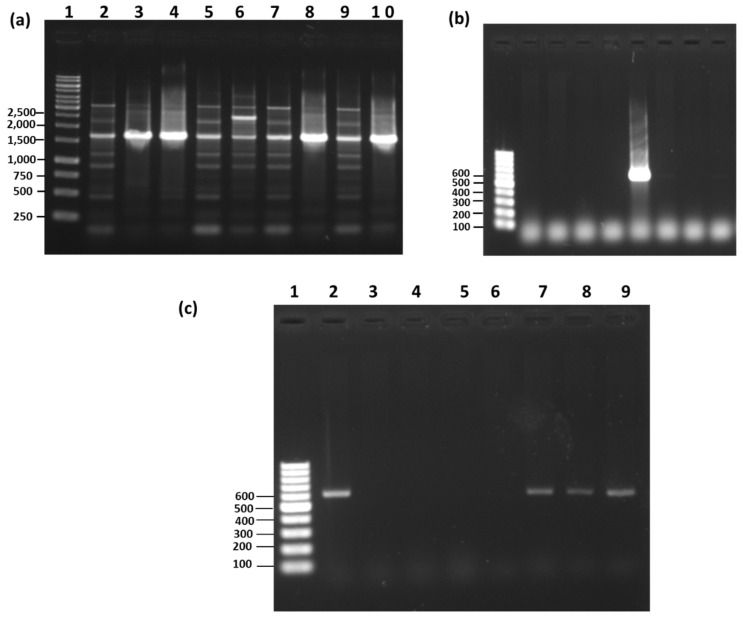
(**a**) RAPD-PCR of various reference strains of *Bacillus licheniformis*. Lane 1: 1 kb size ladder (unit: bp); Lane 2: *Bacillus licheniformis* ATCC21415; Lane 3: *Bacillus licheniformis* KCTC3056; Lane 4: *Bacillus licheniformis* KCTC1029; Lane 5: *Bacillus licheniformis* KCTC1026; Lane 6: *Bacillus licheniformis* KCTC1658; Lane 7: *Bacillus licheniformis* KCTC3559. (**b**) Species-specific PCR (*Blich* gene primer) of pure strains. Lane 1: 0.1 kb size ladder (unit: bp); Lane 2: *Bacillus subtilis* ATCC6633; Lane 3: *Bacillus thuringiensis* KCTC3452; Lane 4: *Bacillus megaterium* KCTC1098; Lane 5: *Bacillus velezensis* KCTC13012; Lane 6: *Bacillus licheniformis* ATCC21415; Lane 7: *Bacillus amyloliquefaciens* KCTC1660; Lane 8: *Bacillus pumilus* ATCC7061; Lane 9: *Bacillus mojavensis* ATCC51516. (**c**) Species-specific PCR using *Blich* gene primer on microorganisms isolated from commercially available organic agriculture materials enriched with effective microorganism. Lane 1: 0.1 kb size ladder (unit: bp); Lane 2: *Bacillus licheniformis* ATCC21415; Lane 3: A-1; Lane 4: B-1; Lane 5: C-1; Lane 6: D-1; Lane 7: E-1; Lane 8: E-2; Lane 9: E-3.

Finally, the isolates E-1, E-2, and E-3, which seem to be *B. licheniformis* by the earlier finding of HiCrome™ *Bacillus* agar, API CHB50, and 16S rRNA gene analysis, were processed for RAPD-PCR against six reference strains of *B. licheniformis* (Figure 7a). The RAPD-PCR suggested that the isolate E-1 (lane 8) and E-3 (lane 10) had a similar RAPD profile to the reference strains, *B. licheniformis* KCTC3056 (lane 3) and *B. licheniformis* KCTC1029 (lane 4) (Figure 7a). In contrast, the RAPD profile of E-2 (lane 9) was similar to that of the reference strains of *B. licheniformis* ATCC21415 (lane 2), *B. licheniformis* KCTC1026 (lane 5), *B. licheniformis* KCTC1658 (lane 6), and *B. licheniformis* KCTC3559 (lane 7) (Figure 7a). Moreover, all the reference strains and E-1, E-2, and E-3 showed *B. licheniformis*-specific dominant amplification at ~1500 bp (Figure 7a). The RAPD profiles of isolates E-1, E-2, and E-3 identified it as *B. licheniformis*. Further, as a confirmatory examination, a species-specific PCR was performed, which amplified ~600 bp fragment only in *B. licheniformis* and not in the other closely related *Bacillus* species (Figure 7b). Consistent with this, isolates E-1, E-2, and E-3 amplified the *B. licheniformis*-specific ~600 bp fragment, confirming that the isolates are *B. licheniformis* (Figure 7c).

## 4. Conclusions

Based on the biochemical and molecular identification methodologies, *B. thuringiensis* and *B. licheniformis* were confirmed in AMEM-D and AMEM-E, respectively. The AMEM-A, AMEM-B, and AMEM-C were tentatively identified as *B. velezensis* based on the findings of 16S rRNA and RAPD-PCR. In conclusion, the findings revealed an accurate identification of *B*. *thuringiensis* in AMEM-D, which supports the claim of the company. Similarly, confirmatory evidence of *B. licheniformis* in AMEM-E was detected. However, for the isolates of A-1, B-1, and C-1, the results were slightly different from the claim of the respective companies and were inclined more towards *B. velezensis* rather than *B. subtilis*. Finally, we conclude that a combination of biochemical tests, staining, and molecular techniques (precisely, species-specific PCR) must be performed to enable the proper identification of closely related species.

## Figures and Tables

**Table 1:** Specification of different commercially available organic agriculture materials enriched with effective microorganism.

Product Code	Product Type	Product Composition	Labelled Microorganisms	pH	Aerobic Bacterial Count
AMEM-A	Liquid	-*Bacillus subtilis* 2% -Extracts of dried ginseng 95%	*Bacillus subtilis*	5.61 ± 0.33	7.92 ± 0.05 Log CFU/mL
AMEM-B	Liquid	-*Bacillus subtilis* 2% -Extracts dried ginseng 60% -Extracts of cinnamon 33%	*Bacillus subtilis*	6.48 ± 0.30	7.80 ± 0.06 Log CFU/mL
AMEM-C	Solid	-*Bacillus subtilis* culture 55.6% -Diatomite 33.4%	*Bacillus subtilis*	6.67 ± 0.15	9.85 ± 0.08 Log CFU/g
AMEM-D	Solid	-Diatomite 58% -*Bacillus thuringiensis* 32%	*Bacillus thuringiensis*	5.52 ± 0.09	9.30 ± 0.02 Log CFU/g
AMEM-E	Liquid	-Fish products 65% -Molasses 20% -Microorganisms 15%	Not specified	3.80 ± 0.16	3.26 ± 0.06 Log CFU/mL

## Supplementary Materials

The following are available online at https://www.mdpi.com/2076-2607/8/10/1568/s1.
