# Supplementary material for: Study on the Identification Methods for Effective Microorganisms in Commercially Available Organic Agriculture Materials
Source: Microorganisms. 2020 Oct 12;8(10):1568. doi: 10.3390/microorganisms8101568 (PMC7599497; doi:10.3390/microorganisms8101568)
Supplement: Supplementary file 1 [file microorganisms-08-01568-s001.pdf]

Supplementary Materials

Study on the Identification Methods for Effective Microorganisms in Commercially Available Organic Agriculture Materials

Ashutosh Bahuguna [1,¶], Ah-ryeong Joe [1,¶], Vishal Kumar [1], Jong Suk Lee [2], Sung-Youn Kim [3], Ji-Young Moon [4], Soon-Kil Cho [3], Hyunjeong Cho [3] and Myunghee Kim [1,*]

[1] Department of Food Science and Technology, Yeungnam University, Gyeongsan, Gyeongsangbuk-do 38541, Republic of Korea; ashubahuguna@ynu.ac.kr (A.B.); whdkfud12@naver.com (A.-r.J.); vkaggarwal180@gmail.com (V.K)

[2] Division of Food & Nutrition and Cook, Taegu Science University, Daegu 41453, Republic of Korea; jslee1213@ynu.ac.kr (J.S.L)

[3] Experiment Research Institute, National Agricultural Products Quality Management Service, Gimcheon 39660, Republic of Korea; youn5326@korea.kr (S-Y.K); skc2224@korea.kr (S-K.C); hjcho201@korea.kr (H.C)

[4] Gyeonggi Provincial Office, National Agricultural Products Quality Management Service, Anyang 14035, Republic of Korea; jymoon76@korea.kr (J-Y.M)

¶ These authors contributed equally to this work.

* Correspondence: e-mail: foodtech@ynu.ac.kr; Tel.: +82-53-810-2958

Table S1. Reference microorganism from different culture collection centers

Species	Strain number	Microorganisms collection organization
Bacillus subtilis	ATCC6633	National Agricultural Products Quality Management Service
	KCTC2217	Korean Collection for Type Cultures
	KCTC3014	Korean Collection for Type Cultures
	KCTC3135	Korean Collection for Type Cultures
	KCTC3239	Korean Collection for Type Cultures
Bacillus thuringiensis	KCTC1508	Korean Collection for Type Cultures
	KCTC1510	Korean Collection for Type Cultures
	KCTC1511	Korean Collection for Type Cultures
	KCTC1524	Korean Collection for Type Cultures
	KCTC1525	Korean Collection for Type Cultures
	KCTC3452	National Agricultural Products Quality Management Service
Bacillus licheniformis	ATCC21415	National Agricultural Products Quality Management Service
	KCTC1026	Korean Collection for Type Cultures
	KCTC1029	Korean Collection for Type Cultures
	KCTC1658	Korean Collection for Type Cultures
	KCTC3056	Korean Collection for Type Cultures
	KCTC3559	Korean Collection for Type Cultures
Bacillus velezensis	KCTC13012	Korean Collection for Type Cultures
	KCTC13417	Korean Collection for Type Cultures
Bacillus amyloliquefaciens	KCTC1660	Korean Collection for Type Cultures
	KCTC1666	Korean Collection for Type Cultures
	KCTC3002	Korean Collection for Type Cultures

Table S2. Details of PCR conditions for species-specific PCR

	***ytcP* gene primer**	***XRE* gene primer**	***Blich* gene primer**
**PCR conditions**	**1)** Initial denaturation (95℃, 5min)<br>**2)** Denaturation (95℃, 30 s)<br>**3)** Annealing (56℃, 1 min)<br>**4)** Extension (72℃, 1min)<br>**5)** Final extension (72℃, 5 min)<br>**6)** PCR cycles (n=30, step 2-4)	**1)** Initial denaturation (95℃, 5 min)<br>**2)** Denaturation (95℃, 30 s)<br>**3)** Annealing (52℃, 30 s)<br>**4)** Extension (72℃, 30 s)<br>**5)** Final extension (72℃, 5 min)<br>**6)** PCR cycles (n=34, step 2-4)	**1)** Initial denaturation (95℃, 5 min)<br>**2)** Denaturation (95℃, 30 s)<br>**3)** Annealing (52℃, 30 s)<br>**4)** Extension (72℃, 30 s)<br>**5)** Final extension (72℃, 5 min)<br>**6)** PCR cycles (n=30, step 2-4)

Table S3. Microscopic characteristics of bacterial isolates from commercially available organic agriculture materials enriched with effective microorganism

Product code	Bacterial isolate code	Microscopic characteristics		
		Gram staining	Cell shape	Spore
AMEM-A	A-1	Positive	Rod	Positive
AMEM-B	B-1	Positive	Rod	Positive
AMEM-C	C-1	Positive	Rod	Positive
AMEM-D	D-1	Positive	Oval rod	Positive
AMEM-E	E-1	Positive	Rod	Positive
	E-2	Positive	Rod	Positive
	E-3	Positive	Rod	Positive

Table S4. API CH50B identification scores of different microbes isolated from commercially available organic agriculture materials enriched with effective microorganism

**Bacterial isolate code**	**Identification results**	**% ID**	**T-index**	**Decision**
A-1	*Bacillus subtilis/ Bacillus amyloliquefaciens*	99.4	0.85	Doubtful profile
	Bacillus licheniformis	0.3	0.58	
B-1	*Bacillus subtilis/ Bacillus amyloliquefaciens*	99.7	0.81	Doubtful profile
	Bacillus megaterium	0.1	0.52	
C-1	*Bacillus subtilis/ Bacillus amyloliquefaciens*	99.4	0.85	Doubtful profile
	Bacillus licheniformis	0.3	0.58	
D-1	*Bacillus cereus*	99.0	0.95	Good identification
	Bacillus mycoides	0.9	0.80	
E-1	*Bacillus licheniformis*	99.9	0.73	Good identification
	Bacillus subtilis/ Bacillus amyloliquefaciens	0.1	0.47	
E-2	*Bacillus licheniformis*	99.7	0.51	Good identification
	Bacillus subtilis/ Bacillus amyloliquefaciens	0.2	0.36	
E-3	*Bacillus licheniformis*	99.7	0.56	Good identification
	Bacillus subtilis/ Bacillus amyloliquefaciens	0.2	0.40	

Table S5. 16S rRNA sequence based identification and comparative analysis of bacterial isolates from commercially available organic agriculture materials enriched with effective microorganism

**Product code**	**Bacterial isolate code**	**Microorganism claimed by AMEM company**	**Identification by 16S rRNA analysis**	**Similarity (%)**
AMEM-A	A-1	*Bacillus subtilis*	- *Bacillus velezensis* CBMB205	99.86
			- *Bacillus subtilis* 168	99.72
AMEM-B	B-1	*Bacillus subtilis*	- *Bacillus velezensis* CBMB205	99.86
			- *Bacillus subtilis* 168	99.72
AMEM-C	C-1	*Bacillus subtilis*	- *Bacillus velezensis* CBMB205	99.86
			- *Bacillus subtilis* 168	99.72
AMEM-D	D-1	*Bacillus thuringiensis*	- *Bacillus proteolyticus* MCCC1A00365	100
			- *Bacillus cereus* ATCC 14579	99.93
			- *Bacillus thuringiensis* ATCC10792	99.79
AMEM-E	E-1	Not specified	- *Bacillus haynesii* NRRL B-1327	97.53
			- *Bacillus licheniformis* DSM13	97.46
	E-2	Not specified	- *Bacillus lichenformis* DSM13	99.93
			- *Bacillus haynesii* NRRL B-41327	99.72
	E-3	Not specified	- *Bacillus haynesii* NRRL B-41327	98.94
			- *Bacillus licheniformis* DSM13	98.87

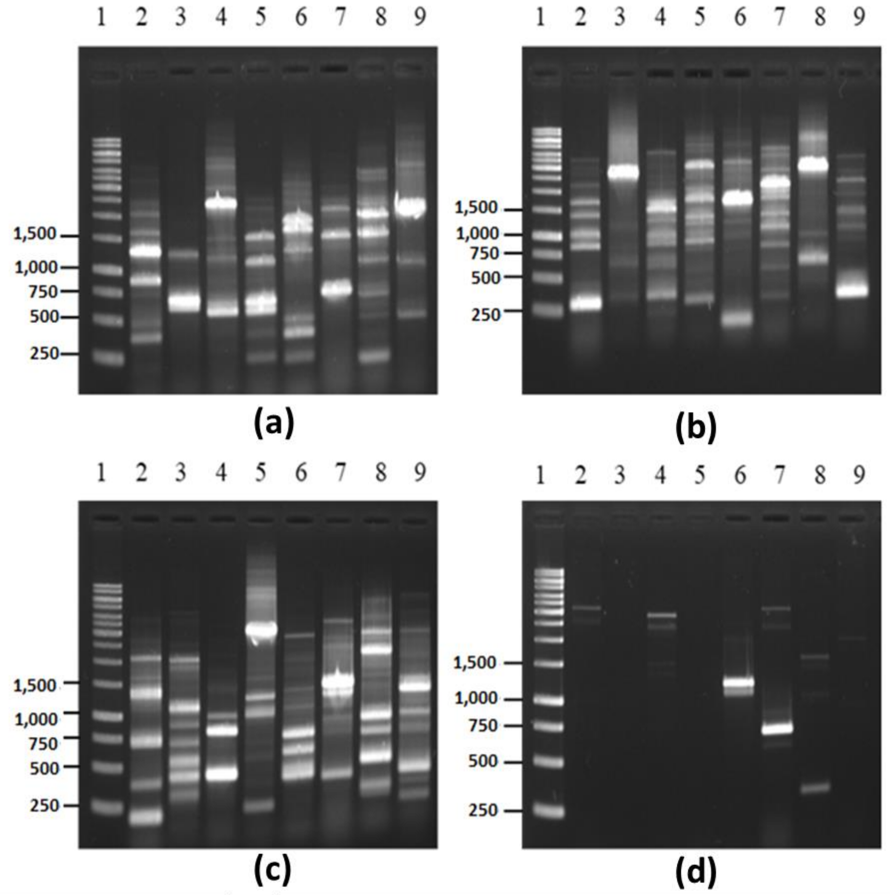

Figure S1. PCR amplified product using various primers. (a): PCR products using primer A; (b): PCR products using primer B; (c): PCR products using primer C; (d): PCR products using primer D. Lane 1: 1 kb size ladder (unit: bp); Lane 2: *Bacillus subtilis*; Lane 3: *Bacillus thuringinesis*; Lane 4: *Bacillus megaterium*; Lane 5: *Bacillus velezensis*; Lane 6: *Bacillus licheniformis*; Lane 7: *Bacillus amyloliquefaciens*; Lane 8: *Bacillus pumilus*; Lane 9: *Bacillus mojavensis*. Agarose gel (1.2%) and primer (2.0 μM) were used for electrophoresis and RAPD-PCR, respectively.

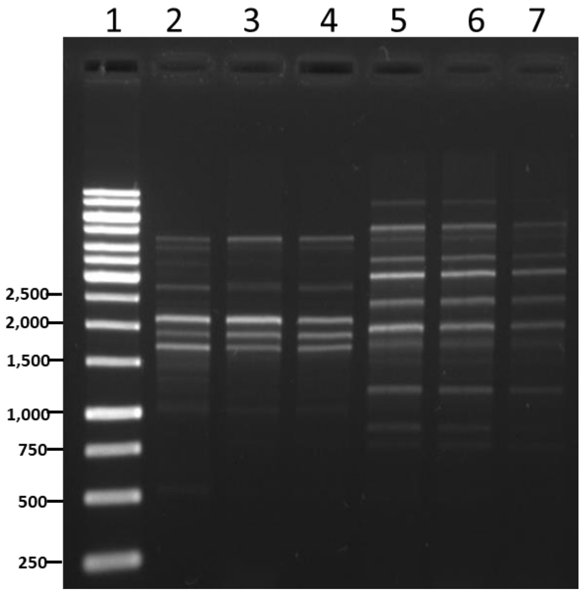

Figure S2. RAPD-PCR of various reference strains of *Bacillus amyloliquefaciens.* Lane 1: 1 kb size ladder (unit: bp); Lane 2: *Bacillus amyloliquefaciens* KCTC1660; Lane 3: *Bacillus amyloliquefaciens* KCTC1666; Lane 4: *Bacillus amyloliquefaciens* KCTC3002; Lane 5: A-1; Lane 6: B-1; Lane 7: C-1.
